# Benzimidazoisoquinoline derivatives inhibit glioblastoma cell proliferation through down-regulating Raf/MEK/ERK and PI3K/AKT pathways

**DOI:** 10.1186/s12935-018-0588-x

**Published:** 2018-06-28

**Authors:** Ya-Jun Zhang, Zhi-Gang Xu, Shi-Qiang Li, Liu-Jun He, Yan Tang, Zhong-Zhu Chen, Dong-Lin Yang

**Affiliations:** Chongqing Engineering Laboratory of Targeted and Innovative Therapeutics, Chongqing Key Laboratory of Kinase Modulators as Innovative Medicine, International Academy of Targeted Therapeutics and Innovation (IATTI), Chongqing University of Arts and Sciences, Chongqing, 402160 China

**Keywords:** Benzimidazoleisoquinolinone, GBM, S phase arrest, Apoptosis, PI3K/Akt pathway, Raf/MEK/ERK pathway

## Abstract

**Background:**

Recent studies showed that benzimidazoleisoquinolinone derivatives exhibit anticancer activity against human cancer cell lines. The aim of this study is to evaluate the anti-tumor effects and mechanisms of benzimidazoleisoquinolinones in isocitrate dehydrogenase-wildtype subtype of human glioblastoma (GBM) cells.

**Methods:**

Human U87 and LN229 cell lines were used to perform the experiments. MTT was applied to screen the effective small molecular inhibitors suppressing growth of GBM cells. Colony formation and BrdU staining assays were performed to assess the inhibition effect of compound-**1H** on the proliferation of GBM cells. The cell cycle and apoptosis were measured by flow cytometry and western blot to analyze the changes of the relative protein expressions and their signal pathways.

**Results:**

Compound-**1H** could suppress GBM cells in a time- and dose-dependent manner. Treatment of compound-**1H** could arrest cell cycle in S phase through up-regulating P21 and P53, and down-regulating cyclin A and E in a dose-dependent manner. Compound-**1H** also induced mitochondrial-dependent apoptosis by increasing Bax, cleaved caspase-3, cleaved caspase-9 and poly ADP-ribose polymerase expression, and decreasing Bcl-2 expression. Moreover, phosphorylated (p)-AKT and p-ERK levels relating to cell proliferation were dramatically decreased in U87 and LN229 cells.

**Conclusions:**

Our results suggest that it is the first time to report the compound-**1H** with benzimidazoleisoquinolinone core playing antitumor activity in human glioblastoma cells by inhibiting Raf/MEK/ERK and PI3K/AKT signaling pathways, and it could be as a lead compound for the further development of targeted glioblastoma cancer therapy.

## Background

Glioblastoma (GBM), which exhibits rapid cellular proliferation, diffuse metastasis and extensive angiogenesis, is the most highly aggressive type of brain tumors in adults [1]. Despite achieved significant advances in GBM research including surgical and pharmacological therapies, malignant gliomas present a unfavorable prognosis accompanied together with serious morbidity and excess mortality [2, 3]. The main obstacle to treatment of GBM is attributed to its high invasiveness characteristics and resistance to radio- and chemo-therapy [4]. Furthermore, it is difficult for surgical treatment to resect all malignant glioma cells which generates high recurrence rate. Therefore, searching for novel drugs and exploring effective strategy seem to be imperative for the treatment of patients with GBM [5, 6].

The ability of selective apoptosis induction is the primary marker for radio- and chemo-therapy, and the aberrant inactivation of apoptosis-related pathways is the major cause of the resistance occurring in malignant glioblastoma [7]. Together with proliferation and differentiation, apoptosis regulates a variety of physiological processes in multifarious tissues. The disruption of homeostatic balances may result in tumorigenesis by excessive cellular proliferation, diffuse migration and many others. Furthermore, the mitogen-activated protein kinase/extracellular signal-regulated kinase (MAPK/ERK) signaling pathway is often abnormally activated in various tumors including glioblastoma, and has become an attractive target for the development of novel anti-neoplastic drugs [8–10]. Therefore, the discovery of potential anticancer agents, which have better bioactivities in inducing apoptosis through the deactivation of MAPK/ERK signaling pathway in glioblastoma cells, seems to be an effective strategy for the drug discovery in glioblastoma research [11, 12].

Benzimidazoisoquinoline structures constitute a wide range of naturally existing products that have various kind of biological activities in the literature, such as an anti-*Trypanosoma cruzi* agent [13], an antibiotic tryptanthrin [14], and a cytotoxic luotonin [15, 16]. Moreover, a small-molecule inhibitor targeted Hsp90 which has been reported to exhibit bioactivity (20 μM) contains a benzimidazoisoquinoline core structure [17]. In an ongoing effort to develop novel and more effective anticancer inhibitors, we have synthetized a series of organic small-molecule compounds based on benzimidazoisoquinoline scaffolds [18]. However, whether these compounds could exert antineoplastic activity against human glioblastoma cells, and what is the feasible mechanism underlying the antigrowth effects in glioblastoma cells, are the central questions addressed by our research.

In this present study, we evaluated the antiproliferative activity of these small-molecule compounds against two different human isocitrate dehydrogenase (IDH)-wildtype subtype glioblastoma cell lines, U87 and LN229, and found that compound-**1H** exhibited better anticancer potential. Moreover, we further analyzed its effects on cell proliferation, apoptosis, and correlative PI3K/AKT and MAPK/ERK signaling pathways, which indicated that the compound-**1H** was possible to be a potential anti-tumor drug-like compound for human GBM-IDH-wt.

## Methods

### Drugs and antibodies

The benzimidazoisoquinoline derivatives were synthesized by Liao et al. as described. The purity of compound-**1H** is more than 95% measured with liquid chromatograph–mass spectrometer (LC–MS) [18]. Compounds were dissolved in dimethylsulfoxide (DMSO) to obtain a 50 mM stock solution, which was then added to the culture medium at a concentration range of 6.25–100 μmol/L. Cells were treated with the compound at indicated concentration for 48 h, and 0.1% DMSO was used as the vehicle. 3-[4,5-dimethylthiazol-2-yl]-2,5-diphenyl-tetrazolium bromide (MTT) were ordered from Sigma-Aldrich. All the primary antibodies and secondary antibodies used in this study were purchased from Cell Signaling Technology.

### Cell lines and culture

Human glioblastoma cell lines U87 and LN229 were purchased from the American Type Culture Collection (ATCC, Manassas, VA, USA). These cells were cultured in high-glucose DMEM (Gibco, USA) supplemented with 10% fetal bovine serum (FBS, Gibco) and 1% penicillin/streptomycin (Gibco) at 37 °C in a humidified incubator containing 5% CO_2_. Both of U87 and LN229 cell lines are isocitrate dehydrogenase (IDH)-wildtype subtype of glioblastoma (GBM-IDH-wt) according to the recent change in classification of gliomas [19]. Moreover, our results obtained in this paper pertain only to the IDH-wildtype subtype.

### Cell viability assay

The antiproliferative activity of compounds was measured with the MTT assay. Briefly, U87 and LN229 cells were seeded into 96-well plates (3000 cells/well) and incubated overnight at 37 °C, then treated with 0, 6.25, 12.5, 25, 50 and 100 μmol/L compounds for 24, 48 and 72 h. Next, 20 μL MTT solution (5 mg/mL) was added into each well and incubated for another 4 h, followed by media removal and solubilization in 200 μL DMSO. The absorbance value was determined at 570 nm using a microplate reader (Bio-Tek, Winooski, VT, USA). Three independent experiments were carried out.

### Brdu staining assay

U87 and LN229 cells were grown in 24-well plate and cultured overnight. After treatment with either DMSO or the compound-**1H** for 48 h, cells were incubated with 10 μg /mL BrdU (Sigma, B5002-100MG, USA) for another 30 min, then fixed in 4% paraformaldehyde (PFA) for 15 min. Followed by the treatment with 1 mol/L HCl and the blockage with 10% goat serum, cells were respectively incubated with primary antibody against BrdU (1:2000, ab6326, Abcam, MA, USA) and Alexa FluorR^®^ 594 secondary antibody for 2 h. DAPI was used for nuclear staining. Finally, the BrdU signal was captured with the Olympus IX73 fluorescence microscope. And then at least 10 microscopic fields were used for the calculation of BrdU percentage.

### Western blot analysis

U87 and LN229 cells were treated with the compound-**1H** (0, 15, 30 and 60 μmol/L) for 48 h, total protein was extracted by lysing the cells in RIPA buffer (CST, Boston, USA) supplemented with protease/phosphatase inhibitor cocktail (CST, Boston, USA) at 4 °C for 30 min. The protein concentration was quantified by the BCA protein assay kit (Beyotime, P0010, Shanghai, China). Protein samples (50 μg) were separated by SDS-PAGE with appropriate gel concentration and subsequent electrophoretically transferred onto PVDF membranes (Millipore Corporation, MA, USA). After blocking with 5% BSA for 2 h at ambient temperature, the membrane sections were incubated gently with the indicated primary antibodies (1:1000, CST, Boston, USA) overnight at 4 °C and washed with TBST for 5 × 5 min. Subsequently, the membranes were then incubated with corresponding HRP-linked secondary antibodies for 2 h. The objective protein signal was obtained by using the ECL reagent (GE Healthcare, RPN3244, USA) and the chemiluminescence detection instrument (Tannon 5200 Multi, China).

### Cell cycle analysis

U87 and LN229 cells were grown in 100 mm cell culture dishes for 24 h and then treated with the indicated compound-**1H** concentrations for 48 h. For cell cycle assay, after harvested by trypsinization and washed with cold PBS, adherent cells were fixed in 70% ethanol at 4 °C for 24 h. Subsequently, the fixed cells were washed twice and stained with cycle test system containing 200 μL PBS, 1 μL propidium iodide (1 mg/mL) and 1 μL RNase (10 mg/mL) at 37 °C for 20 min in the dark. The samples were detected using a FACS C6 (Becton Dickinson, San Jose, CA) and the data were obtained by analyzing with FlowJo7.6 software. Every experiment was carried out in triplicate, 50,000 events per sample were recorded.

### Cell apoptosis assay

Apoptosis induced by the compound-**1H** in U87 and LN229 cells was analyzed with a FACS using the Annexin V-FITC/PI apoptosis kit (BD, 556547). Briefly, after 48 h of the compound-**1H** treatment, U87 and LN229 cells were harvested and then resuspended with 1× binding buffer to a cell density of 1 × 10^6^ cells/mL. Subsequently, the cells were stained with 5 μL Annexin V-FITC and 5 μL PI (50 μg/mL) and then incubated in the dark room for 15 min. The stained cells were diluted with 1× binding buffer to appropriate density, and immediately detected using FACS. The percentage of apoptotic cells was obtained using FACS.

### Hoechst 33342 staining assay

Nuclear fragmentation was monitored by Hoechst 33342 with staining apoptotic nuclei. U87 and LN229 cells were treated with various concentrations of compound-**1H** for 48 h, then the cells were harvested, washed twice with cold PBS and fixed with 4% paraformaldehyde (PFA) for 15 min at room temperature. The fixed cells were then washed and stained with 5 μg/mL Hoechst 33342 for 30 min. Digital images were captured with Olympus IX73 fluorescence microscope. Finally, at least 10 microscopic fields were used for the calculation of nuclear percentage.

### Mitochondrial membrane potential (∆Ψm) assay

The difference of mitochondrial membrane potential was monitored using the ∆Ψm-specific fluorescent probe Rhodamine 123 (Sigma-Aldrich, USA) as previously described [20]. Briefly, after treatment with different concentrations of compound-**1H**, U87 and LN229 cells were harvested and washed twice with cold PBS. Then the cells were incubated with Rhodamine 123 for 30 min at 37 °C in the dark, and relative fluorescence intensities of the samples were analyzed using flow cytometry with setting of FL1A at 530 nm and FL2H 585 nm. Each experiment was carried out in triplicate, and the results were expressed as the mean ± SD.

### Soft agar colony formation assay

Colony formation ability was measured with the soft agar assay on U87 and LN229 cells. Briefly, for base agar, 1 mL DMEM complete medium containing 0.6% low-melting agarose was added into a 6-well plate. After solidity of 0.6% base agar, U87 and LN229 cells (about 1 × 10^3^ cells) were mixed with 1 mL DMEM complete medium and 0.3% low-melting agarose, and then were laid on the base layer to form a top agar. The compound-**1H** was added to both base layer and top layer. After 14–20 days of treatment, cells were stained with 300 μL MTT in each well and incubated at 37 °C in the dark room for 20 min. In the end, the stained colonies in each well were photographed and the number of colonies with more than 50 cells was counted manually.

### Statistical analysis

Each datum point was expressed as mean ± SD of three independent experiments. Statistical analysis between different groups was performed using the GraphPad Prism 5 software program. The value of P < 0.05 (indicated by *) between two groups was deemed to be statistically significant.

## Results

### Compound-**1H** reduces glioblastoma cell proliferation and viability

**Fig. 1:**
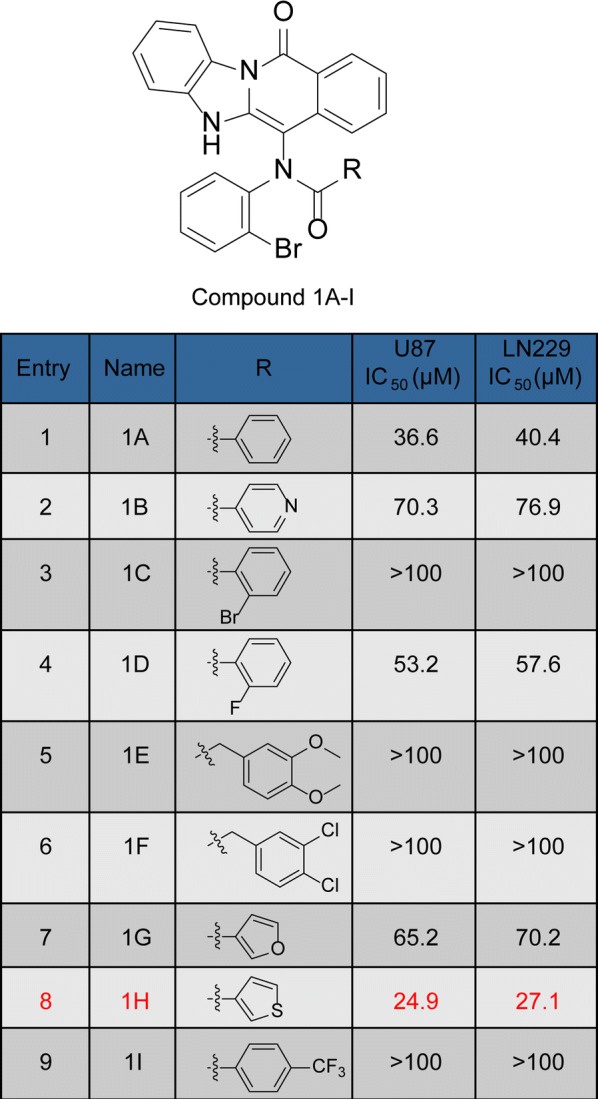
Antiproliferation effects of benzimidazoisoquinoline derivatives in human glioblastoma cells. Chemical structures of the synthetic benzimidazoisoquinoline derivatives were labeled by compounds **1A**–**I**. IC_50_ values were also obtained after 48 h with treatment of compounds **1A**–**I** at concentrations of 3.125–50 μmol/L in U87 and LN229 cells and represented as percentage of control as mean ± SD of experiments performed at least three times

**Fig. 2:**
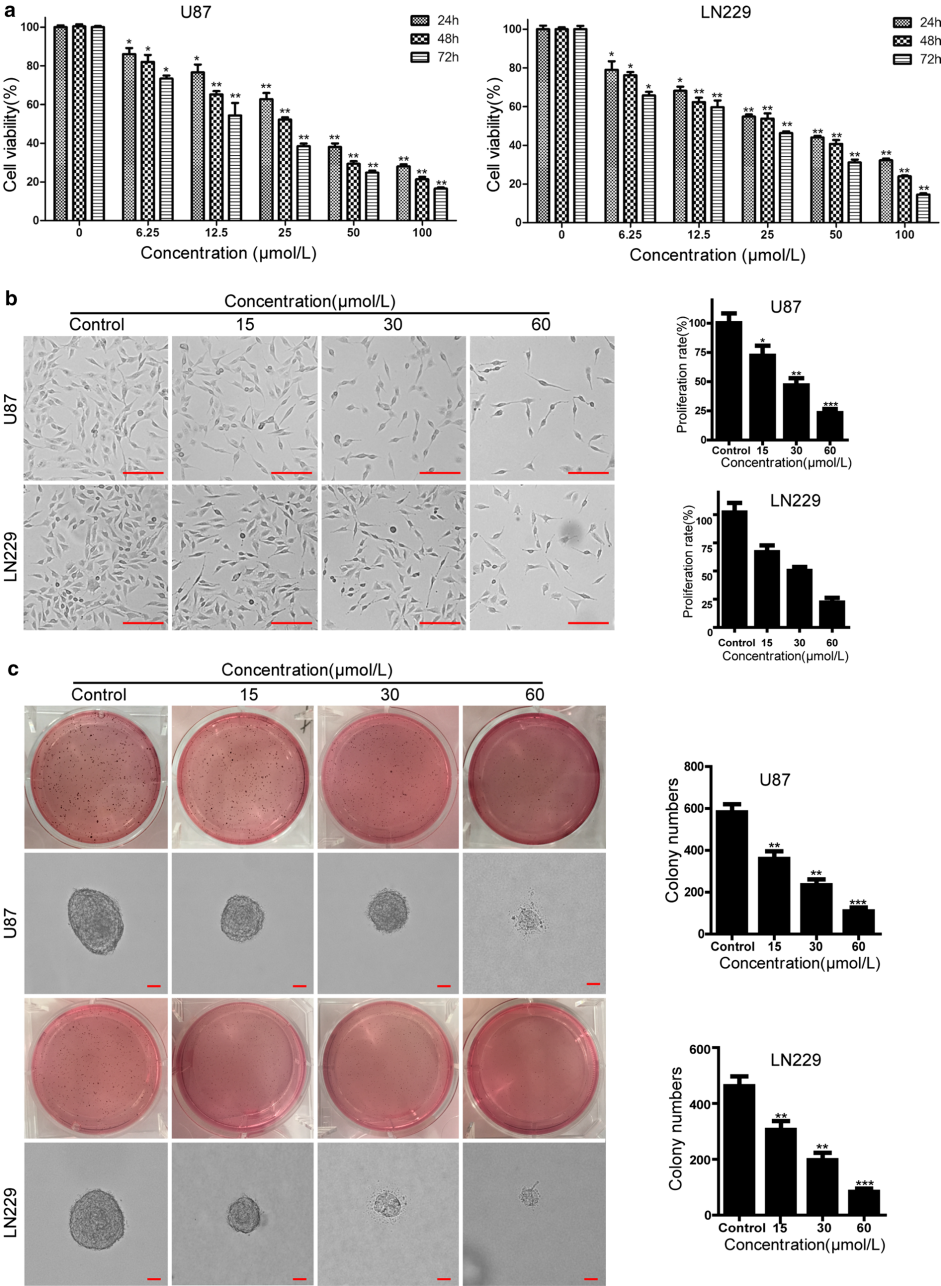
Compound-**1H** reduces cell proliferation and viability of human glioblastoma cells. **a** U87 and LN229 cells were treated with the different concentrations of compound-**1H** for 24, 48 and 72 h. Cell viability was measured with the MTT assay. **b** Cell morphology of U87 and LN229 glioblastoma cells was captured with microscope after treating with vehicle (0.05% DMSO) or the indicated concentrations of compound-**1H** for 48 h. Scale bar, 100 μm. The histogram showed the quantification of cell proliferation rate. **c** The soft agar assay was employed to detect colony formation in vitro after treating with the indicated concentrations of compound-**1H** for 14 days. The colonies were visualized with the images and quantitated by histogram. Scale bar, 100 μm. All data were demonstrated as the mean ± SD of three independent experiments. *P < 0.05; **P < 0.01; ***P < 0.001 versus vehicle

**Fig. 3:**
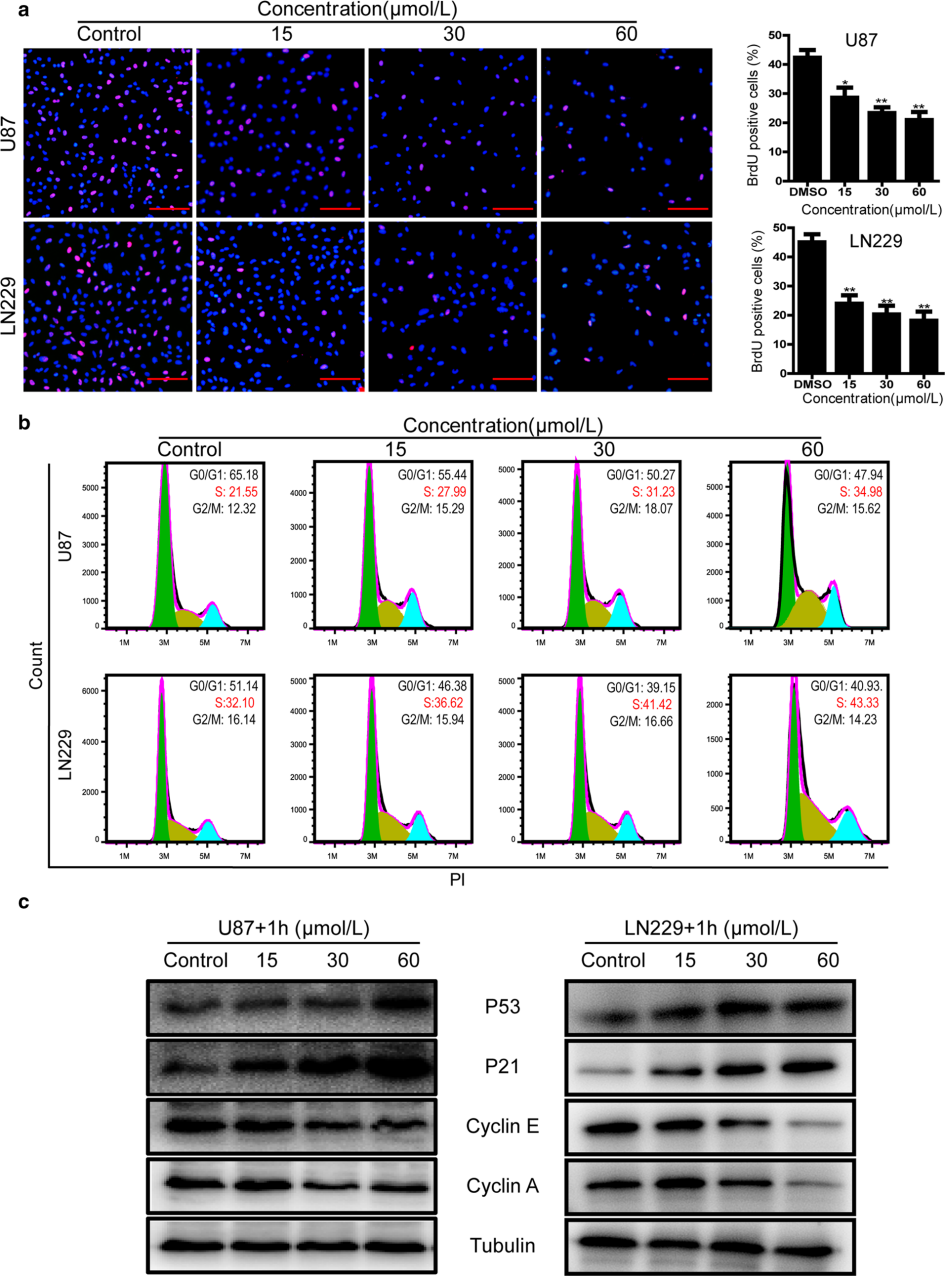
Compound-**1H** induces cell cycle arrest at S-phase to suppress glioblastoma cell proliferation. **a** Proliferation and quantification of U87 and LN229 cells were determined by BrdU staining assay with the treatment of compound-**1H** for 48 h. Scale bar, 100 μm. **b** The cell cycle of U87 and LN229 cells was measured by flow cytometry in the presence of vehicle or compound-**1H**, and the percentages of cell population in different periods were quantitated with three independent experiments. **c** Effects of compound-**1H** on the expression levels of cyclin A, cyclin E, P21 and P53 in U87 and LN229 cells were determined by western blot, with *β*-tubulin as a loading control. All data were demonstrated as the mean ± SD of three independent experiments. *P < 0.05; **P < 0.01; ***P < 0.001

Given the anticancer effects of the benzimidazoisoquinoline scaffold, we have synthetized 9 organic small-molecule compounds in our previous report [18]. To evaluate the antiproliferative activity against human glioblastoma cells, their IC_50_ values were detected using MTT. MTT result indicated that compound-**1H** exhibited better anticancer potential than compound-**1A**–**1G** and **1I**. The IC_50_ values in U87 and LN229 cells were 24.92 and 27.12 μmol/L, respectively (Fig. 1). As indicated in Fig. 2a, the compound-**1H** efficiently inhibited glioblastoma cell viability in a time- and dose-dependent manner. Besides, the cell counting assay with the microscope showed that glioblastoma cell proliferation was observably reduced by the compound-**1H** (Fig. 2b). Moreover, soft agar assay was performed to evaluate the effect of the compound-**1H** in colony formation, and the results demonstrated that smaller and lesser colonies were formed in treated groups (15, 30 and 60 μmol/L) compared with control groups in both glioblastoma cell lines (Fig. 2c). Also, BrdU staining assay analysis in U87 and LN229 cells showed that compound-**1H** induced a prominent decrease in the percent of BrdU-positive cells in both cell lines (Fig. 3a). These results supported that the compound-**1H** dramatically inhibited cell viability and proliferation in human glioblastoma cells.

### Compound-**1H** induced S-phase cell cycle arrest in human glioblastoma cells

To gain an insight into the mechanism underlying antiproliferative activity of the compound-**1H** in human glioblastoma cells, cell cycle analysis was performed in U87 and LN229 cells. As shown in Fig. 3b, the results obtained by flow cytometry demonstrated that the compound-**1H** induced cell cycle arrest at S phase in U87 and LN229 cells. Representative histograms further indicated that the percentage of S-phase cells significantly increased (from 21.55 to 34.98% for U87 cells, P < 0.01; from 32.10 to 43.33% for LN229 cells, P < 0.01). To further confirm the results, western blot was performed to detect the levels of the S-phase related proteins, and found that the compound-**1H** treatment decreased the levels of Cyclin A and Cyclin E, and however, increased the levels of P21 and P53 in a dose-dependent manner (Fig. 3c). Taken together, these results suggested that the compound-**1H** inhibited cell proliferation by inducing S-phase cell cycle arrest.

### Apoptotic effects of the compound-**1H** on human glioblastoma cells

**Fig. 4:**
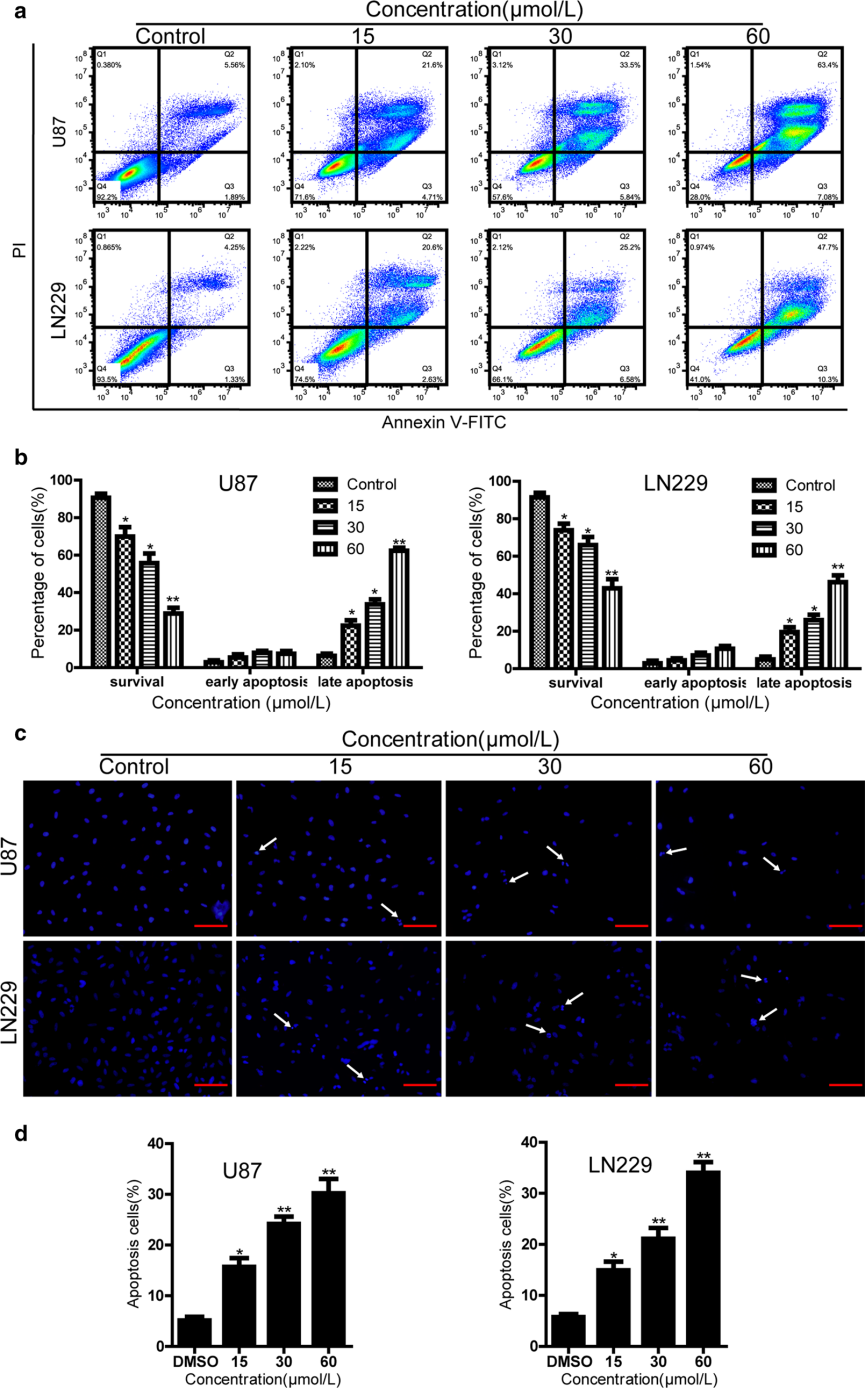
Compound-**1H** induces apoptosis in human glioblastoma cells. U87 and LN229 cells were exposed to various concentrations of compound-**1H** for 48 h. **a** Cells treated with compound-**1H** were stained with annexin-V/PI. The Q4 (annexin-V−/PI−), Q3 (annexin-V+/PI−) and Q2 (annexin-V+/PI+) quadrants represent the populations of normal, early apoptotic and late apoptotic cells, respectively. **b** Percentages of surviving cells, early and late apoptotic cells are shown as the mean ± SD (n = 3). **c** Apoptotic nuclear morphological changes induced by compound-**1H** (0, 15, 30, 60 μmol/L) for 48 h were observed using Hoechst 33342 staining in U87 and LN229 cells. The arrowheads indicate apoptotic cells which exhibit highly condensed and fragmented nuclear morphologies. **d** Percentages of apoptotic cells are shown as the mean ± SD (n = 3). *P < 0.05, **P < 0.01, ***P < 0.001 compared with the control group

**Fig. 5:**
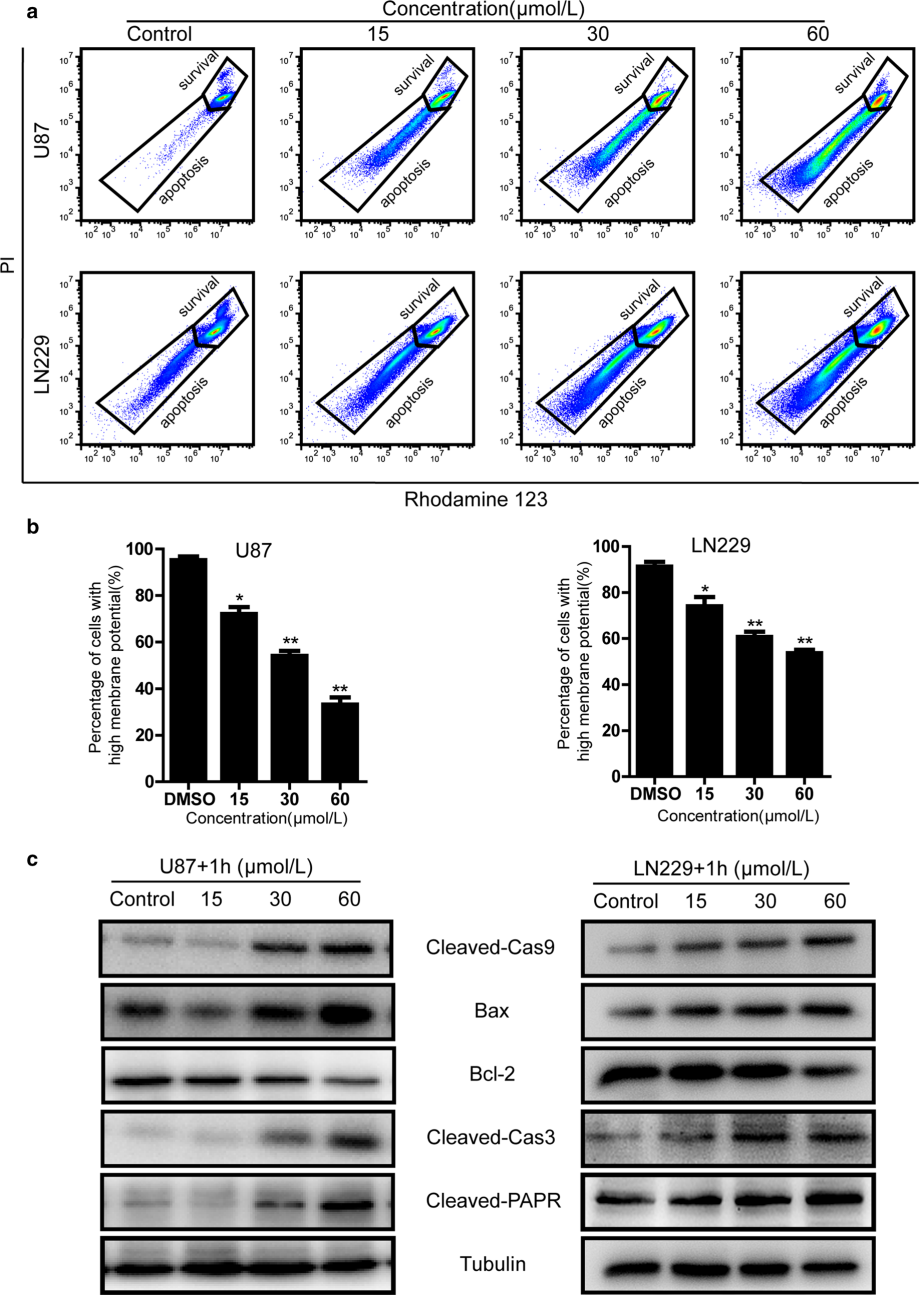
Compound-**1H** induces the mitochondria-mediated intrinsic apoptosis via the regulation of apoptosis-related proteins in human glioblastoma cells. U87 and LN229 cells were treated with the compound-**1H** (0, 15, 30, 60 μmol/L) for 48 h. **a** Flow cytometry analysis of ∆Ψm by Rhodamine 123 staining. **b** The corresponding histogram shows the percentages of cells with high ∆Ψm (survival) and low ∆Ψm (apoptosis). Values represent the mean ± SD (n = 3). *P < 0.05; **P < 0.01; ***P < 0.001 compared with the control group. **c** After treatment with the compound-**1H** (0, 15, 30, 60 μmol/L) for 48 h, cell lysates of U87 and LN229 cells were prepared, and the expression of cleaved caspase-9, Bax, Bcl-2, cleaved caspase-3 and cleaved PARP were analyzed using western blot. *β*-Tubulin was used as a loading control

To further investigate whether the antiproliferative effects of compound-**1H** was caused by the induction of apoptosis, an Annexin V-FITC/PI assay was employed via flow cytometry after U87 and LN229 cells exposed to the compound-**1H** for 72 h. The typical images and histograms in Fig. 4a, b indicated that the compound-**1H** dramatically induced cellular apoptosis in both cells and the proportion of early- and late-phase apoptosis were significantly increased (from 5.56 to 63.4% for U87 cells, P < 0.01; from 4.25 to 47.7% for LN229 cells, P < 0.01) in a dose-dependent manner. Furthermore, the results of Hoechst 33342 staining assay revealed that typical apoptotic cells which were characterized as the condensed and fragmented nuclei were more easily observed in the compound-**1H** treatment groups rather than the vehicle group (Fig. 4c, d). To examine whether the mitochondrial membrane integrity was damaged by compound-**1H** treatment, Rhodamine 123 was employed to measure ∆Ψm in the compound-**1H** treated glioblastoma cells. Compared with the control group, compound-**1H** treatment induced a dose-dependent decrease in ∆Ψm (Fig. 5a, b). We found that more than 50% of U87 cells and 30% of LN229 cells showed are induction in ∆Ψm after treatment with 60 μmol/L compound-**1H** for 48 h. Furthermore, western blot assay was performed to confirm the apoptotic effects of the compound-**1H**. As shown in Fig. 5c, glioblastoma cells treated with the compound-**1H** led to an elevation of apoptosis-related proteins (Bax, cleaved caspase-3, cleaved caspase-9 and cleaved PARP), while Bcl-2 was down-regulated in a dose-dependent manner. In summary, the apoptotic effect induced by compound-**1H** relies on the activation of apoptosis-related proteins through a mitochondrial-dependent apoptotic pathway in human glioblastoma cells.

### Inhibition effects of the compound-**1H** on AKT and ERK signaling pathways in human glioblastoma cells

**Fig. 6:**
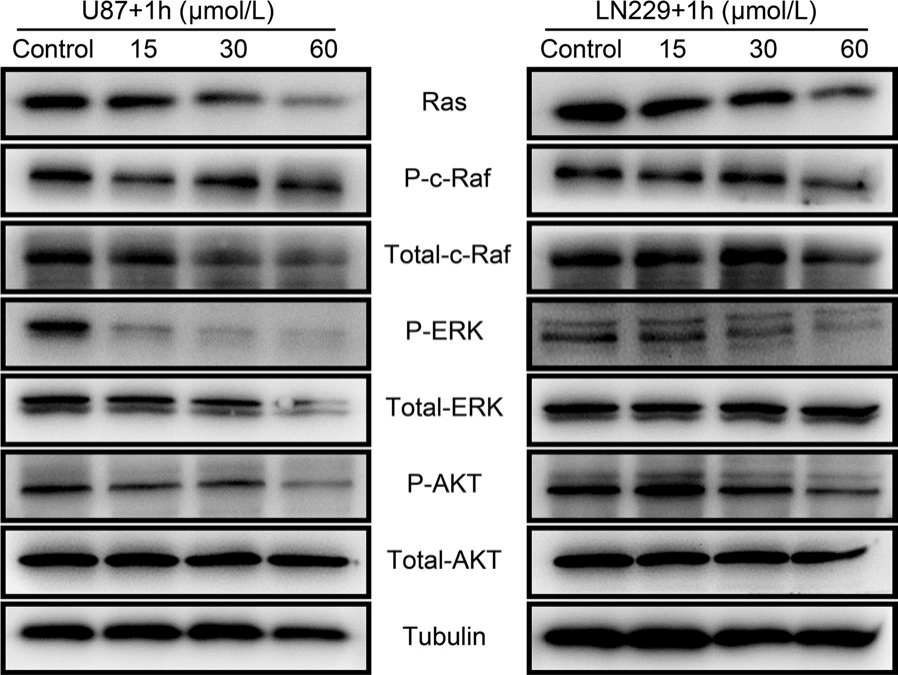
Compound-**1H** inhibited AKT and ERK signaling pathways in human glioblastoma cells. U87 and LN229 cells were treated with different concentrations (0, 15, 30 and 60 μmol/L) of the compound-**1H** for 48 h. The expression levels of Ras, c-Raf, P-Raf, Akt, P-Akt, ERK, and P-ERK were determined by western blot. β-Tubulin was used as a loading control. The results were representative of three independent experiments

Accumulating research has indicated that the signaling pathways of PI3K/AKT and Ras/MAPK/ERK, which are associated with various protein kinase cascades, contribute to tumor growth in malignant glioblastoma [8, 9, 12]. Hence, to further clarify whether the antitumor action of compound-**1H** was mediated through these pathways in U87 and LN229 cells, the related proteins of AKT and ERK pathways were examined by western blot in the compound-**1H**-treated cells. As shown in Fig. 6, treatment with the compound-**1H** in a concentration dependent manner for 48 h resulted in the effective inactivation of MAPK/ERK signaling pathway through the significant phosphorylation inhibition of c-Raf and ERK. Furthermore, AKT phosphorylation at Ser-473 was similarly suppressed in U87 and LN229 cells, which revealed that AKT signaling pathway may also participate in the regulation of the inhibition effects upon treatment with the compound-**1H**. In conclusion, our findings indicated that both AKT and ERK signaling pathways play a vital role in the compound-**1H**-induced inhibition effects against human glioblastoma cells.

## Discussion

Accumulating evidence has certified that benzimidazoisoquinoline scaffolds have attracted considerable attention due to their broad bioactivities existence in both natural products and chemosynthetic inhibitors [18]. As a consequence, it is imperative to acquire novel molecules containing benzimidazoisoquinoline cores and assess their anticancer effects. In our earlier study, a series of benzimidazoisoquinoline derivatives have been synthetized. This research evaluates antiproliferative activity of these compounds against human GBM-IDH-wt cells and provides an interesting lead compound-**1H**, which prompts us to further investigate its antigrowth efficiency and mechanism in U87 and LN229 cell lines. However, the effect of this compound on glioblastoma—IDH-mutant is unknown.

In this study, we found that the compound-**1H** remarkable inhibited GBM-IDH-wt cell proliferation and clonogenesis ability in a dose-dependent manner, confirming the inhibitory efficacy of the compound-**1H** on glioblastoma cells. Furthermore, the experiment of cell cycle distribution measured with flow cytometry demonstrated that treatment with the compound-**1H** induced a significant cell cycle arrest at S-phase in U87 and LN229 cells, which prompted us to further explore the feasible mechanism underlying the arrest of cell cycle progression. The regulation of cell cycle is relevant to many proteins such as CDKs, Cyclins, P21 and P53. Indeed, results of western blot assays indicated that the compound-**1H** downregulated the levels of Cyclin A and Cyclin E whereas upregulated the levels of P21 and P53 in both U87 and LN229 cells. Based on these results, we believe that the compound-**1H** arrests cell cycle in S-phase to exert its antiproliferation activity in human GBM-IDH-wt cells.

Apoptosis, known to be abnormally regulated in various cancers, is considered as a primary defense mechanism against tumorigenesis [21, 22]. Numerous anti-cancer chemicals execute their inhibitory efficiency by the induction of apoptosis, which is mediated by two major pathways: the death-receptor-induced extrinsic pathway and the mitochondria-mediated intrinsic pathway [23–25]. It is widely believed that either the activation of caspase-8 caused by extrinsic pathway or the activation of caspase 9 induced by intrinsic pathway can eventually activate caspase 3, an indispensable terminal caspase, which is capable of cleaving downstream cellular substrates, such as PARP, leading to apoptosis progression. Moreover, the anti-apoptotic and pro-apoptotic members of the Bcl-2 protein family, such as Bcl-2 and Bax, play an imperative role in the mitochondrial apoptosis pathway to mediate the activation of downstream caspases [26, 27]. Compound-**1H** treatment of U87 and LN229 cells induced activation of caspase 9 and caspase 3, concurrently cleavage of PARP, demonstrating that apoptotic effects of the compound-**1H** were associated with the mitochondrial pathway. In addition, the corresponding protein expression of Bcl-2 and Bax are observed, suggesting regulation of Bcl-2 family proteins contributed to the apoptosis progression. Ultimately, the inhibitory efficiency of the compound-**1H** relies on the induction of apoptosis in human glioblastoma cells.

The signaling pathways involving Raf/MEK/ERK and PI3K/AKT, characterized by frequent deregulation in various cancer types, are closely associated with tumorigenesis via the regulation of several cellular progressions such as cellular proliferation and apoptosis [28–30]. Currently, accumulating evidence supports that the ERK and AKT signaling pathways contribute to the promotion of proliferation and the prevention of apoptosis in human glioblastoma cells [31, 32]. Furthermore, several valid inhibitors targeting the components of ERK and AKT signaling pathways have shown potential clinical benefits against various malignant tumors including glioblastoma [33–36]. In the present study, our findings imply that ERK and AKT signaling pathways were dramatically inhibited by the compound-**1H** in U87 and LN229 cells, as indicated by the down-regulated expression of Ras, c-Raf, p-Raf, p-ERK and p-AKT. Therefore, results of our study have discovered feasible molecular mechanisms underlying the anti-glioblastoma effect of the compound-**1H**, although the detailed upstream event requires further investigation.

## Conclusions

Our present study verifies that the compound-**1H** exhibits significant anti-proliferation efficiency in U87 and LN229 cells by inducing cell cycle arrest at S-phase. The compound-**1H** has the potential to induce apoptosis by up-regulating Bax, cleaved caspase-9, cleaved caspase-3, and cleaved PARP and down-regulating Bcl-2 protein levels in glioblastoma cells. Furthermore, the investigation indicates that the compound-**1H** exerts its anti-glioblastoma efficiency through significant inhibition of Raf/MEK/ERK and PI3K/AKT signaling pathways, which accelerates our comprehension on the molecular mechanisms of the compound-**1H** in human glioblastoma cells. Hence, our study illuminates the molecular basis of the compound-**1H**, which may contribute to its potential applications for the valid treatment of GBM-IDH-wt.

## Abbreviations

GBM: glioblastoma
CST: cell signaling technology
DMEM: Dulbecco’s modified eagle medium
FBS: fetal bovine serum
PBS: phosphate buffer saline
DMSO: dimethyl sulfoxide
PFA: paraformaldehyde
MTT: 3-(4,5-dimethyl-2-thiazolyl)-2,5-diphenyl-2-*H*-tetrazolium bromide
Brdu: 5-bromo-2′-deoxyuridine
DAPI: 4′,6-diamidino-2-phenylindole
FACS: fluorescence activating cell sorter
FITC: fluorescein isothiocyanate
PI: propidium iodide
RIPA: radio immunoprecipitation assay
SDS-PAGE: sodium dodecyl sulfate polyacrylamide gel electrophoresis
PVDF: polyvinylidene difluoride
TBST: tris buffered saline with Tween-20
ECL: electro-chemiluminescence system
BSA: bovine serum albumin
SD: standard deviation
Ras: rat sarcoma
RAF: rapidly accelerated fibrosarcoma
ERK: extracellular regulated protein kinases
MAPK: mitogen-activated protein kinase
CDKs: cyclin-dependent kinases
BAX: Bcl-2 associated X protein
BCL-2: B-cell lymphoma-2
PARP: poly ADP-ribose polymerase
PI3K: phosphatidyl inositol 3-kinase
AKT: protein kinase B
DNA: deoxyribonucleic acid
IC_50_: 50% inhibitory concentration
